# A Comparison between Emergency Medicine Residency Training Programs in the United States and Saudi Arabia from the Residents' Perception

**DOI:** 10.1155/2014/362624

**Published:** 2014-01-19

**Authors:** Khaled Alghamdi, Abdulaziz Alburaih, Mary Jo Wagner

**Affiliations:** Emergency Medicine Residency Program, Central Michigan University College of Medicine, Saginaw, MI, USA

## Abstract

*Objectives*. This study was designed to compare the trainees' perception of emergency medicine (EM) training in the United States (US) and Saudi Arabia (SA) and to identify residents' levels of confidence and points of satisfaction in education, procedural skills, and work environment. *Method*. An IRB-exempt anonymous web-based survey was distributed to five EM residency training programs in the USA and three residency regions in SA. *Results*. 342 residents were polled with a 20% response rate (16.8% USA and 25.8% SA). The Saudi residents responded less positively to the questions about preparation for their boards' examinations, access to multiple educational resources, and weekly academic activities. The Saudi trainees felt less competent in less common procedures than US trainees. American trainees also more strongly agree that they have more faculty interest in their education compared to the Saudi trainees. The Saudi residents see more patients per hour compared to their US peers. *Conclusion*. These findings may be due to the differences in training techniques including less formal didactics and simulation experience in SA and more duty hour regulations in the USA.

## 1. Introduction

There is an increasing need for emergency medicine (EM) development and EM trained physicians throughout the world. EM in Saudi Arabia (SA) is based on the North American model and is considered a new specialty which has nonetheless grown dramatically in the last decade [1]. The United States (US) was one of the first countries to establish and recognize EM as a specialty and many countries use this system as a model for EM training. Because EM training is a relatively new endeavor in Saudi Arabia, our aim is to evaluate and compare Saudi and US residents' level of confidence and satisfaction with their training from trainees' perspectives. To our knowledge, there are no similar studies reported in the literature, and no studies compare residency training systems between countries. This research contributes to the literature by addressing this gap.

## 2. Materials and Methods

### 2.1. Study Design

The survey was designed to query the residents on many of the standards for U.S. EM residency training and other important topics. We used many of the questions from the Council of Emergency Medicine Residency Directors (CORD) survey and the Accreditation Council for Graduate Medical Education (ACGME) residents' survey to establish our survey. The survey was divided into 3 sections: education, clinical skills, and work environment with a total of 17 questions. The study was reviewed by the institutional review board and was determined to be exempt from needing study consent.

The education section asked residents about topics such as adequate preparation for written and oral board exam, the incorporation of evidence-based medicine into practice, the efficient use of diagnostic studies, access to multiple educational resources, whether the program devotes at least four hours per week to academic activity, the benefit and relevance of weekly academic activities, program preparation to manage multiple trauma patients simultaneously, and preparation to manage critically ill pediatric and adult patients.

The clinical skills section asked residents to provide information about experiences with competency in performing procedures (i.e., bedside ultrasound, emergency thoracotomy, thoracotomy, basic and advanced airway management, pericardiocentesis, vaginal deliveries, procedural sedation, orthopedic reduction and dislocation, central line placement, cardiac pacing, pediatric airway management, and resuscitation) and about their experiences with simulation training.

Finally, in the work environment section they were asked about topics such as faculty member supervision, bedside teaching, level of interest by faculty and staff in resident education, opportunities for faculty evaluation, and number of patients seen.

### 2.2. Methods

This was a cross-sectional, multi-institutional study that anonymously surveyed Saudi and US EM residents as a convenience sample of residents who agreed to complete the survey. The survey was maintained on an online secure server at our institution. No identifying information, including IP address, was collected from participants. Participants were recruited by sending the survey webpage link to Saudi residents through email contacts. In the USA, program directors were contacted via email and asked to distribute the survey among US residents in five residency programs. The survey was distributed in January and February 2013 to five residency training programs in the USA (Central Michigan University, University of Maryland, Baylor College of Medicine, George Washington, and Emory University) and three residency programs in Saudi Arabia (eastern, western, and central regions). The study group included only the residents who spent at least six months in EM residency training. Five reminders were sent to each group during the two-month period.

## 3. Results

Of the 342 surveys distributed to residents in both the US and Saudi residency programs, the overall response rate was 21.3% (16.8% USA and 25.8% SA). The response rate by level of training was 61.8% from junior and midlevel residents (PGY 1-2) and 38.2% from senior residents (PGY 3-4). The survey was divided into three main sections, education, clinical skills, and work environment, as detailed above. Percentages of residents in the USA and SA answering that they agree or strongly agree with survey questions are presented in Table 1.

### 3.1. Education

**Figure 1:**
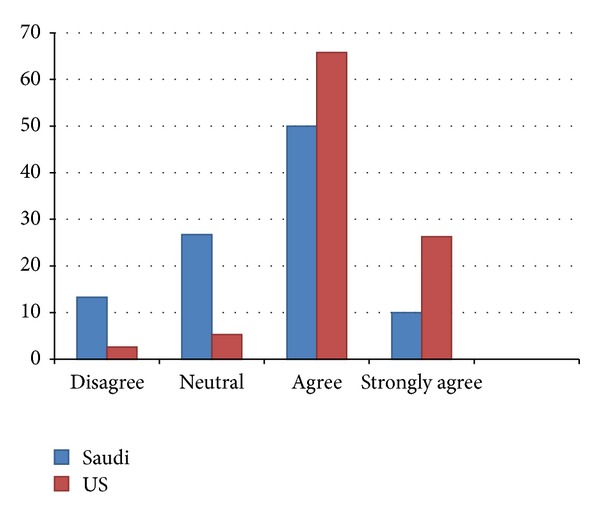
The response was mostly negative when Saudi residents were asked if the residency program adequately prepared them for the written and oral board exam.

In general, residents in the USA are more likely to agree that the educational aspects of their programs are adequately training them for practice and that there is sufficient academic environment for training, with many of these differences being statistically greater (Table 1). For instance, residents in the USA were more likely to agree or strongly agree that their programs are adequately training them for board exams (see Figure 1) and are preparing them to manage multiple trauma patients, that their academic activities are beneficial and relevant, and that there is easy access to educational resources. In only one area (preparing to manage critically ill pediatric patients) do SA residents rate their program higher than in the USA, but this difference is not statistically significant.

### 3.2. Clinical Skills

There are also several areas where US residents were more likely to agree or strongly agree that their program provides opportunities for competency in clinical skills (Table 1). These skills include cricothyrotomy, pericardiocentesis, vaginal deliveries, and cardiac pacing. Although there were several clinical skills where 100% of residents in both groups indicated that their programs provided opportunities for competency, in general, again, US residents were more likely to agree or strongly agree that their programs provided opportunities for skills competency when compared to SA residents. Interesting, only 36.7% of SA trainees indicate that they have access to a simulation lab, while 100% of US residents said they have access to a simulation lab. All US and SA trainees thought that having access to a simulation lab would prepare them better to perform procedures.

### 3.3. Work Environment

Regarding work environment, SA residents reported seeing more patients per hour compared to US residents. Approximately the same percentage of US and SA residents and the same percentage of each group reported that faculty and staff were at least “slightly interested” in resident education. However, US residents were more likely to report that faculty was very or extremely interested in education compared to SA residents (92.1% versus 51.7%). Finally, greater numbers of US residents, compared to SA residents, report being able to evaluate their faculty on an annual basis (100% versus 66.7%).

## 4. Discussion

Emergency medicine represents a relatively new specialty, and the United States was one of the first countries to recognize and establish emergency medicine residency training programs. Most of its developmental history has occurred in the last three decades or so. EM development as a specialty in most other developed countries was based upon by the US model [1]. The establishment of EM training in Saudi Arabia was in the year 2000 by newly graduated physicians who had completed their EM training in North America [2].

Forty percent of Saudi residents have indicated that their training program was not adequately providing preparation for the oral and written board exam. A neutral response was considered negative response since preparation for the board exams was felt to be one of the primary goals of a specialty training program. This lack of training may be influenced by the lack of easy access to multiple educational resources and the perception of the Saudi residents that their faculty has little interest in residents' education.

An interesting finding of the study was the statistically increased number of patients per hour seen by the Saudi residents compared to their US peers. The newer duty hour restrictions and supervision guidelines in the USA may limit the average experience of the more junior residents in the USA and thus decrease the average patients/hour ratio. It is unclear how important the total number of supervised patient encounters is to a resident's success in achieving competency. It has been suggested that the combination of a certain number of patient encounters with the use of deliberate practice in caring for patients provides the education to develop competency and expertise. In addressing this, the Saudi training programs provide more patient care experience for their trainees according to this survey.

A significant majority of Saudi trainees felt that their training program did not provide adequate educational opportunity to become competent with performing uncommon procedures (such as cricothyrotomy, pericardiocentesis, cardiac pacing, and vaginal deliveries). This may be explained by the limited availability of simulation labs (63.3% of Saudi residents have no access to simulation labs). This could also explain the high neutral response when Saudi residents were asked about managing multiple trauma patients and the relevance of weekly academic activity to their daily clinical practice. Two recent meta-analyses across all clinical disciplines (including EM) and one meta-analysis for EM learners have demonstrated that, when compared with no intervention or baseline performance, involvement in simulation has a large effect on the outcomes of knowledge, skills, and behavior of a trainee and moderate effects on patient-related outcomes of the participating residents [3–5].

Residents' evaluation and feedback of their faculty is considered one of the important elements of the ongoing quality improvement of medical training in the USA Successful programs use this as a tool to understand and listen to trainee's perspective. Compared to the US group, fewer Saudi residents are able to evaluate their faculty at least once per year. The culture of evaluation and feedback may be vastly different for the Saudi residents and this may not be a central element in their residency training system.

The response rate remains an important limitation of the study. However, as the first study of its kind, this initial data may still provide some areas for discussion and comparison for further studies in the future. Language and cultural differences may also limit the comparison of EM residency programs between the two countries.

As emergency medicine residency training progresses in Saudi Arabia, a few areas might provide improvement in the residents' perception of their training. These include implementation of fully equipped simulation laboratories in EM residency program with opportunities for the trainees to perform uncommon procedures and conduct resuscitation simulation in managing difficult cases. In addition, it appears that there should be an increase in the resident accessibility to multiple educational resources including text books, ebooks, journals, visual media, and reference materials. It is also believed that faculty development could be increased to enhance faculty interest, educate the faculty on bedside teaching, and share teaching tools and feedback processes. This might be done through local and regional programs as well as affiliation with international emergency medicine educational organizations such as CORD.

## 5. Conclusion

US trainees reported feeling more competent in managing multiple traumas and in certain procedural skills compared to SA trainees. Both faculty supervision and faculty interest in education were felt to be less strong by SA residents. Saudi residents see more patients per hour compared to US residents. These findings may be due to the differences in training techniques including less formal didactics and simulation experience in SA and more duty hour regulations in the USA A few changes including the inclusion of simulation in the EM curriculum in Saudi Arabia might increase the Saudi residents' opinion of their residency training experience.

## Figures and Tables

**Table 1:** Percent of residents answering agree or strongly agree to survey items.

Survey question	% answering agree or strongly agree		*P* value
	US Residents	SA Residents	
Education			
Residency program is adequately training me for written/oral board exams	92.1%	60.0%	<0.05
Residency program is adequately preparing me to incorporate EBM in my practice	97.4%	90.0%	NS
Residency program is preparing me to competently manage critically ill patients	100.0%	100.0%	NS
Residency program is preparing me to competently manage multiple trauma patients simultaneously	97.4%	83.3%	<0.05
Residency program is preparing me to competently manage critically ill pediatric patients	73.7%	79.3%	NS
Residency program is teaching me to utilize diagnostic studies	97.4%	90.0%	NS
My residency program devotes at least 4 hours/week to academic activity	100.0%	100.0%	NS
Weekly academic activities are beneficial and relevant to daily clinical practice	97.4%	83.3%	<0.05
Easy access to multiple educational resources	100.0%	69.0%	<0.05
Clinical skills			
Program provides opportunities for competency in bedside ultrasound	100.0%	93.3%	NS
Program provides opportunities for competency in emergency thoracotomy	100.0%	100.0%	NS
Program provides opportunities for competency in thoracostomy	100.0%	100.0%	NS
Program provides opportunities for competency in intubation	100.0%	100.0%	NS
Program provides opportunities for competency in difficult airway procedure	100.0%	80.0%	NS
Program provides opportunities for competency in nasotracheal intubation	52.6%	42.3%	NS
Program provides opportunities for competency in cricothyrotomy	89.5%	51.7%	<0.05
Program provides opportunities for competency in intraosseous infusion	100.0%	93.3%	NS
Program provides opportunities for competency in pericardiocentesis	78.9%	48.3%	<0.05
Program provides opportunities for competency in vaginal deliveries	97.4%	70.0%	<0.05
Program provides opportunities for competency in procedural sedation	97.4%	100.0%	NS
Program provides opportunities for competency in orthopedic reduction/dislocation	92.1%	100.0%	NS
Program provides opportunities for competency in pediatric intubation	81.1%	76.7%	NS
Program provides opportunities for competency in pediatric resuscitation	100.0%	93.3%	NS
Program provides opportunities for competency in central line placement	100.0%	100.0%	NS
Program provides opportunities for competency in cardiac pacing	92.1%	73.3%	<0.05
Residency program has a simulation lab (answered “yes”)	100.0%	36.7%	<0.05
Work environment			
No. of patients/hour (answered “>2 patients/hour”)	2.6%	56.7%	<0.05
How sufficient is faculty supervision? (Ranked “slightly sufficient or above”)	100.0%	96.6%	NS
How interested are faculty and staff in your residency education? (Ranked “at least slightly interested or above”)	100.0%	96.6%	NS
Ranked “very interested or extremely interested”	92.1%	51.7%	<0.05
Are able to evaluate faculty at least once per year (answered “yes”)	100.0%	66.7%	<0.05
